# Effects of High-Temperature Stress on Biological Characteristics of *Coccophagus japonicus* Compere

**DOI:** 10.3390/insects15100801

**Published:** 2024-10-14

**Authors:** Ying Sun, Meijuan Yang, Zhengpei Ye, Junhong Zhu, Yueguan Fu, Junyu Chen, Fangping Zhang

**Affiliations:** 1 Environment and Plant Protection Institute, Chinese Academy of Tropical Agricultural Sciences, Haikou 571101, China; sy15263434847@163.com (Y.S.); meijuany@163.com (M.Y.); zhengpeiyecn@163.com (Z.Y.); fygcatas@163.com (Y.F.); jychencn@163.com (J.C.); 2 College of Plant Science and Technology, Huazhong Agricultural University, Wuhan 430070, China; 3 School of Tropical Agriculture and Forestry, Hainan University, Haikou 570228, China; 4 Hainan Provincial Engineering Research Center for the Breeding and Industrialization of Natural Enemies, Haikou 571101, China; 5 Sanya Research Institute of Chinese Academy of Tropical Agricultural Sciences, Sanya 572025, China

**Keywords:** *Coccophagus japonicus* Compere, *Parasaissetia nigra* Nietner, high-temperature stress, development, reproduction, parasitism function

## Abstract

**Simple Summary:**

This study reveals that exposure to short-term high temperatures of 36 °C, 38 °C, and 40 °C, or continuous high temperatures of 32 °C and 34 °C, can adversely affect the growth, development, reproduction, and parasitism of *Coccophagus japonicus* Compere. In addition to negatively affecting adult female insects, *C. japonicus* larvae and pupae exposed to both short-term and continuous high-temperature stress showed impaired survival rates, delayed development, reduced longevity, and decreased fecundity, which also impacted offspring survival and parasitism. The results suggest that the degree of temperature stress and its duration play crucial roles in parasitoid development.

**Abstract:**

The parasitoid, *Coccophagus japonicus* Compere (Hymenoptera: Aphelinidae) is a dominant natural enemy of *Parasaissetia nigra* Nietner (Hemiptera: Coccidae), an important pest of rubber trees. Much of Chinese rubber is cultivated in hotter regions such as Yunnan and Hainan, exposing applied parasitoids to non-optimal temperatures. Therefore, *C. japonicus* must adapt to avoid temperature-related impacts on survival and population expansion. In this study, we monitored the survival rate, developmental duration, parasitism rate, and fecundity of *C. japonicus* during short-term exposures to 36 °C, 38 °C, and 40 °C for 2, 4, and 6 h, as well as continuous exposures to 32 °C and 34 °C for 3 days. The results show that short-term exposure to high-temperature stress leads to decreased survival rate of *C. japonicus* larvae and pupae, with survival rates declining as temperature and duration increase. High-temperature stress also delayed insect development, reduced mature egg production, shortened the body length of newly emerged females, and decreased female lifespans. Moreover, continuous high-temperature stress was found to significantly impact the development and reproduction of *C. japonicus*. Compared with the CK (27 °C), 3 d of continuous exposure to 34 °C prolonged developmental duration, shortened the body length and lifespan of newly emerged females, reduced survival rate and single female fecundity, and significantly decreased offspring numbers and parasitism rates. Temperatures of 36 °C, 38 °C, and 40 °C decreased the mortality time of adult females to 28.78, 16.04, and 7.91 h, respectively. Adverse temperatures also affected the insects’ functional response, with 8 h of stress at 36 °C, 38 °C, and 40 °C causing the control efficiency of *C. japonicus* on *P. nigra*. This level of stress in the parasitoids was found to reduce the immediate attack rate and search effect, prolong processing time, and attenuate interference between small prey. Parasitoid efficiency was lowest following exposure to 40 °C. In this study, we determined the range of high temperatures that *C. japonicus* populations can tolerate under short- or long-term stress, providing guidance for future field applications.

## 1. Introduction

Insect life processes are greatly influenced by temperature [1], with exposure to heat beyond the thermophilic zone adversely affecting survival, development, reproduction, behavior, and physiology [2]. Furthermore, heat stress can impair the hunting and parasitizing abilities of parasitic and predatory insects [3,4], thereby altering their ecological roles. In high-temperature regions, even minor variations in average temperature can significantly exacerbate the negative effects experienced by insects. Insects living in tropical regions are inevitably subjected to heat stress during at least one life stage, with impacts closely linked with the intensity and duration of temperature exposure [5]. The strength and frequency of heat waves are predicted to increase due to the ongoing threat of global climate change [6,7], potentially altering critical trophic levels and disrupting the overall structure and function of various ecosystems. Extreme heat can cause dehydration, impair intracellular ion concentrations, disrupt neural transmission, weaken the synthesis of biological macromolecules, and impede system functions [8,9]. These effects ultimately trigger changes in insect physiological responses and their underlying mechanisms. For instance, heat stress in *Drosophila melanogaster* Meigen induces recessive mutations in the DNA, which may increase genetic diversity but cause an immediate fitness penalty [10]. Among the genetic effects of heat stress, a change in the expression levels of transposases and methylases has also been demonstrated. The increase in transpositions generates gene variability, essential for adaptation [11]. In contrast, *Hippodamia variegate* Goeze and *Propylaea quatuordecimpunctata* Linnaeus increase the expression of Heat Shock Protein 70 (Hsp70), which defends against cellular heat damage [12]. These adaptations to high temperatures are beneficial to insect growth and development, enabling them to withstand changing climates.

The soft-scale insect, *Parasaissetia nigra* Nietner (Hemiptera: Coccidae), has emerged as a significant pest of rubber trees in tropical regions, causing immense damage in China since its initial outbreak in Yunnan in 2004 [13]. *Coccophagus japonicus* Compere (Hymenoptera: Aphelinidae) is the dominant parasitoid of *P. nigra*, with a parasitism rate as high as 77.1%, offering an effective method for pest control in agricultural settings [14,15]. The wasp also targets many species of coccidaes, such as *Ceroplastes floridensis* Comstock, *Ceroplastes rubens* Maskeel, *Coccus hesperidum* Linnaeus, *Ceroplastes japonicas* Green, *Coccus pseudomagnoliarum* Kuwana, and *Saissetia oleae* Olivier [16]. As an endoparasitoid, *C. japonicus* parasitizes the second and third instar larvae, as well as early adults of *P. nigra*, with the highest emergence rate observed in third instar larvae [17]. Temperature has been found to significantly affect the survival, development, and reproduction of this parasitoid, with optimal control against *P. nigra* occurring within the range of 24–27 °C [14,15,18]. Previous research has documented the effects of low temperatures on *C. japonicus*, reporting that the emergence rate of 3-day-old pupae was 88.00% after 27 d at 12 °C but decreased to 44.67% at 10 °C [19]. Another study revealed that the survival rates, body length of newly emerged female wasps, mature egg loads, and adult female lifespans were positively correlated with increasing temperatures during short-term exposure to 2 °C, 4 °C, and 6 °C. However, these parameters exhibited a negative correlation with the duration of the stress, ranging from 0.5 to 2 d. Additionally, the developmental period of the insects was inversely proportional to the temperature of the stress and directly proportional to the length of exposure [20].

While the effects of low and optimal temperatures on *C. japonicus* have been investigated, little is known about the parasitoid’s responses to high temperatures. Further elucidation of this relationship is imperative for understanding and predicting the resilience of biological systems in the face of a warming planet [21,22,23,24,25,26]. Given that *P. nigra* predominantly inhabits tropical and subtropical regions, pest control efforts through field applications of *C. japonicus* may be hindered by the inevitable heat stress [27]. Given the prevalent high temperatures in Hainan, we have designed a series of varying temperatures and durations to systematically evaluate the physiological responses of the *C. japonicus* to high temperature stress. In this study, we investigated the growth and reproduction of *C. japonicus* larvae and pupae after short-term (36 °C, 38 °C, and 40 °C for 2, 4, and 6 h) and long-term (32 °C and 34 °C for 3 d) high-temperature stress in an indoor setting. For each treatment, we recorded the survival rate of adult *C. japonicus*, developmental period, body length of newly emerged females, number of mature eggs, and adult female lifespans. We also monitored the survival rates of adult females and the effects on parasitism following exposure to 36 °C, 38 °C, and 40 °C stress. Our results provide empirical evidence for the viability of *C. japonicus* as a pest control method in agricultural settings.

## 2. Materials and Methods

### 2.1. Insect Collection and Preparation

The initial population of *P. nigra* was collected from an experimental farm at the Yunnan Institute of Tropical Crops in Yunnan Province, China (21.95°N, 101.45°E). Subsequent generations were reared on mature pumpkin fruit in the insect breed room of the Environment and Plant Protection Institute, Chinese Academy of Tropical Agricultural Sciences (CATAS) in Hainan Province, China, under controlled laboratory conditions (26 ± 1 °C, 70–90% RH). The original population of *C. japonicus* was collected from the Danzhou Experimental Base (19.51°N, 109.49°E) at the Institute of Environment and Plant Protection, CATAS. Offspring were reared in a laboratory setting, with *P. nigra* serving as both a substrate for egg-laying and a food source for developing larvae [20]. Both *P. nigra* and *C. japonicus* populations belong to the Environment and Plant Protection Institute, CATAS.

To assess the effects of temperature stress, two mated two-day-old parasitoid females were placed into a cylindrical oviposition device. The device is a homemade transparent plastic container featuring a diameter of 7.5 cm and a height of 8.5 cm, with a 1 cm diameter aperture at its base. The device was positioned over approximately 30 third-instar scale larvae and placed in a climate chamber with standard conditions (27 ± 1 °C, 70–80% RH, 12L: 12D) (MGC-350HP-2, Shanghai Blue Pard Co., Ltd., Shanghai, China) to allow for parasitism. The parasitoids and the oviposition device were removed following 24 h. The parasitized scale larvae were maintained on whole pumpkins in the chamber to enable development.

To assess the effects of temperature stress on *C. japonicus*, samples were collected at various stages. This includes the third instar stage (approximately 10 d after oviposition), the three-day-old pupal stage (parasitoid prepupae visible through the abdomen), the fully developed pupal stage, and the adult stage (newly emerged females). All experimental samples were carefully selected to ensure good health and uniform sizing.

### 2.2. Short-Term High-Temperature Stress Assays

The survival, development, and reproduction of *C. japonicus* were evaluated under various short-term high-temperature stress treatments during larval and pupal stages. Sixty *P. nigra*, in which *C. japonicus* developed to the third instar, were used as larval samples for the experiment. Additionally, 30 three-day-old *C. japonicus* pupae were also collected as the pupal samples. The larval and pupal samples were placed in climate chambers at 36 °C, 38 °C, and 40 °C for 2, 4 and 6 h. Following stress treatment, samples were transferred into a climate chamber maintained at 27 ± 1 °C for continued culturing. In addition to these nine treatments, another suit of scale larvae and pupae described above were reared continuously in a climate chamber with standard conditions (27 ± 1 °C, 70–80% RH, 12L:12D) as the control group.

Once they developed a brown body color, *C. japonicus* pupae were removed from the pumpkins and placed in test tubes (12 mm × 60 mm). The survival rate and development duration (days until adulthood) were continuously monitored. Upon emergence, every five new females were collected into one test tube, provided with 20% sucrose water as a food source and cultured in a climate chamber (27 ± 1 °C). The 20% sucrose solution is 20 g sucrose using ddH2O volume to 100 mL. Survival rates were recorded every 24 h until all samples perished and longevity was calculated. The body length of newly emerged females was measured using a stereomicroscope (JSZ8, Nanjing Jiangnan Yongxin Op-tics Co., Ltd., Nanjing, China). To assess egg-laying by stressed parasitoids, the female and male parasitoids were placed in a 1:1 ratio for one day to ensure successful mating. Two mated females were placed in a petri dish (a diameter of 9 cm) with 30 third-instar scale larvae, which were replaced every 24 h until the parasitoids died. Each parasitized insect was dissected under a stereomicroscope to record the total number of eggs deposited and calculate the oviposition rate. In each experimental group, the fecundity of six parasitoids and the lifespan of 30 wasps were observed, and the experiment was replicated three times.

### 2.3. Continuous High-Temperature Stress Assays

The survival, development, and reproduction responses of *C. japonicus* to continuous high-temperature stress during larval and pupal stages were investigated by placing samples in climate chambers set at 32 °C and 34 °C. Following 3 d of stress treatment, the samples were transferred to a climate chamber maintained at 27 ± 1 °C for continued culturing. The control larvae and pupae groups were consistently reared at 27 ± 1 °C.

To determine the effects of continuous high temperature on *C. japonicus* offspring, 60 third-instar *P. nigra* larvae and four two-day-old females *C. japonicus* reared in high-temperature stresses were placed in an oviposition device and incubated in a 27 ± 1 °C climate chamber. These two-day-old females were allowed to mate for one day before the experiment. After 24 h, the female adults and the device were removed, while the parasitized scale insects were left for further development. Once the new generation of *C. japonicus* developed to the brown pupal stage, they were placed in test tubes and maintained in climate chambers. The number of hatchlings, developmental duration, body length of newly emerged females, and parasitism rate were recorded. The experiment was replicated three times.

### 2.4. Survival and Biological Control Assay Following High-Temperature Stress

The effects of high-temperature stress on the biological control functions of *C. japonicus* were examined by calculating the lethal time and parasitism function of treated insects. Thirty similarly sized newly emerged *C. japonicus* females were collected in test tubes and placed in climate chambers under the following conditions: 36 °C for 8, 16, 24, 32, or 40 h; 38 °C for 8, 12, 16, 20, or 24 h; 40 °C for 4, 6, 8, 10, or 12 h; and 27 ± 1 °C constant temperature for the control. Insects were supplied with 20% sucrose water, and adult survival rates and lethal durations were recorded. The experiment was replicated three times.

The impact of temperature on parasitism was examined by placing individual pairs of male and female parasitoids in test tubes for one day to allow mating. Male and female insects were separated and transferred to climate chambers set to 36 °C, 38 °C, and 40 °C for 8 h. After 1 h recovery at 27 ± 1 °C, the females were placed in individual Petri dishes containing pumpkins with 5, 10, 15, 20, 25, and 30 third-instar larval scale insects. Adult females were removed after 24 h of incubation, and parasitism rates were calculated by dissecting the hosts under a stereomicroscope. The experiment was replicated three times.

### 2.5. Data Processing

SPSS 23.0 was used for experimental data analysis, while Microsoft Excel 2021 was used for chart generation. The Scheirer–Rey–Hare test was used to analyze the effects of various temperatures and stress durations on key life parameters of *C. japonicus* larvae and pupae, including survival rate, development period, body length of newly emerged females, mature egg number, and female adult longevity. Survival rate data were adjusted using inverse sine square root transformation and the number of mature eggs was logarithmic transformed before analysis of variance. Probit regression was used to analyze the median lethal time (LT50) of female *C. japonicus* adults. One-way Analysis of Variance (ANOVA) was employed for all remaining parameters, followed by Duncan’s multiple range test for comparing the differences in means.

Holling-II functional response model [28]: *Na = a′NT*/(1 + *a′ThN*), *Na* is the number of parasitized *P. nigra*. *a′* is the instantaneous attack rate. *N* is the density of *P. nigra*. *Th* is the average amount of time it takes to parasite *P. nigra*. And *T* is the total time of discovery and parasitism of *P. nigra* by *C. japonicus*.

## 3. Results

### 3.1. Effects of Short-Term High-Temperature Stress on C. japonicus

Short-term exposure to high-temperature stress during both pupal and larval stages significantly influenced the survival, development, and reproduction of *C. japonicus*. Both temperature and the duration of stress heavily impacted life parameters; however, they elicited slightly different physiological reactions. As the temperature stress increased, the survival rate, body length, mature egg mass of newly emerged female adults, and lifespan of *C. japonicus* decreased, whereas the duration of development was relatively increased. Variations in stress duration conferred similar effects on the parasitoids, though it did not notably impact female body length or mature egg mass in the larval treatment group, nor did it affect female life span in the pupal treatment group (Table 1 and Table 2).

Exposure to 4 or 6 h at 36 °C, 38 °C, and 40 °C, and 2 h at 38 °C and 40 °C during the larval stage significantly reduced the survival rate of *C. japonicus* compared to the CK (control). Developmental duration was significantly delayed, and female body length was reduced when parasitoid larvae were subjected to 38 °C for 6 h or 40 °C stress for more than 4 h. Moreover, increases in temperature and stress duration led to a gradual reduction in adult female lifespan and the number of mature eggs (Table 3).

During the pupal stage, exposure to 36 °C for 6 h or 38 °C and higher for any duration significantly impaired *C. japonicus* survival rate compared to the CK. Developmental durations were delayed, and female body length was reduced when parasitoid pupae experienced 40 °C stress for more than 4 h and 2 h, respectively. Exposure to 36 °C for 4h or 6 h, as well as all durations of 38 °C, significantly reduced the number of mature eggs. Finally, any exposure above 38 °C at the pupal stage significantly decreased adult female lifespan compared to the CK (Table 4).

### 3.2. Effects of Continuous High-Temperature Stress on C. japonicus

The temperature and duration of continuous high-temperature stress during the larval and pupal stages significantly influenced the survival, development, and reproduction of *C. japonicus* (Table 5, Table 6, Table 7 and Table 8).

When *C. japonicus* larvae were maintained at 34 °C for 3 d, the survival rate (F = 15.450, *p* = 0.004, *n* = 381), developmental duration (F = 38.990, *p* < 0.001, *n* = 381), and body length of newly emerged female wasps (F = 10.111, *p* = 0.012, *n* = 90) were significantly lower than those kept at 32 °C and the CK.

Larvae exposed to 32 °C and 34 °C for 3 days exhibited reductions in female longevity (F = 38.802, *p* < 0.001, *n* = 291) and female fecundity (F = 65.020, *p* < 0.001, *n* = 18) when compared to the CK. No significant differences were observed between the other treatment groups (Table 5). Additionally, continuous high temperatures at the larval stage affected the number of emerged offspring, with significantly lower numbers observed in groups exposed to 34 °C stress for 3 d compared to those kept at 32 °C and the CK (F = 4.5, *p* = 0.064, *n* = 372). Compared to the CK, sustained exposures to temperatures over 32 °C did not notably impact the developmental duration (F = 2.517, *p* = 0.161, *n* = 372), body length (F = 1.583, *p* = 0.280, *n* = 90), and parasitism rates (F = 1.344, *p* = 0.329, *n* = 18) of offspring (Table 7).

In pupal treatment groups, continuous exposure to temperatures over 32 °C led to reductions in the survival rate, with declines becoming more pronounced at higher temperatures. Developmental duration (F = 111.938, *p* < 0.001, *n* = 255) was significantly reduced following 3 d of stress at 32 °C but increased after 3 d at 34 °C. Although a decrease in the body length of newly emerged females was observed, this difference was not significant. Compared to the CK, continuous exposure to heat stress above 32 °C resulted in reductions in female longevity (F = 113.107, *p* < 0.001, *n* = 255), with effects exacerbated by increasing temperatures. Additionally, when parasitoid pupae endured 34 °C stress for 3 d, single female fecundity (F = 82.788, *p* < 0.001, *n* = 18) was significantly decreased compared to the CK (Table 6). Continuous exposure to temperatures exceeding 34 °C led to progressively reduced numbers of emerged offspring and parasitism rates. When parasitoid pupae experienced 34 °C heat stress for 3 d, the number of emerged offspring (F = 25.590, *p* < 0.001, *n* = 326) and their parasitism rate (F = 8.887, *p* = 0.016, *n* = 18) declined significantly (F = 111.938, *p* < 0.001, *n* = 255). However, the developmental duration (F = 1.669, *p* = 0.265, *n* = 326) and body length (F = 0.001, *p* = 0.999, *n* = 90) of the offspring did not differ significantly from CK (Table 8).

### 3.3. Effects of High-Temperature Stress on the Biological Control Function of C. japonicus

The median lethal time (LT50) of treated *C. japonicus* decreased with increasing temperature, with LT50 values of 28.78 h, 16.04 h, and 7.91 h at 36 °C, 38 °C, and 40 °C, respectively. These findings demonstrate a gradual decrease in insect heat tolerance as temperatures rise (Table 9).

When *C. japonicus* was exposed to 36–40 °C for 8 h, the parasitism rate tended to stabilize at approximately 20–30 scales. Following 8 h of stress at 36 °C, parasitism rates were significantly lower than CK, when scale density exceeded 15. Significant differences were also observed in temperature treatments over 38 °C, with the lowest number of *P. nigra* parasitized by *C. japonicus* at 5.67 in the 40 °C treatment. At the same stress temperature, the number of parasitized scales increased as scale density rose from 5 to 30. This finding indicates that stress temperature and scale density influence parasitism by *C. japonicus* (Table 10).

The Hollings-II model was employed to fit the parasitism data (Table 10). The *R*^2^ values for all parameters were greater than 0.9856, meeting the significance criteria. The *χ*^2^ values ranged from 0.1629 to 1.1899, and a chi-square test confirmed that *χ*^2^ was less than *χ*^2^ _(0.05)_ = 11.07, indicating consistency between the theoretical and observed values. When stressed at 36–40 °C, *C. japonicus* took longer to parasitize the scale insects compared to CK. Following 8 h of treatment, the immediate attack rate, parasitism efficiency, and parasitism upper limit were negatively correlated with temperature increases. The upper limit of parasitism at 40 °C was 1/6 of the CK value, indicating that high-temperature stress greatly reduced the parasitism efficiency in *C. japonicus* (Table 11).

## 4. Discussion

The results of this study support the notion that short-term exposure to high temperatures exerts detrimental impacts on the growth, development, survival, and reproduction of *C. japonicus* [1]. Our experiments reveal that temperature and stress duration exert a cumulative dose effect on the *C. japonicus* survival rate, with increases in both variables exacerbating the adverse effects of heat stress. These results align with a previous study on the parasitoid *Trichogrammatoidea bactrae* Nagaraja, which observed that increasing pupal exposure to high-temperature stress reduced adult survival and emergence rates while increasing malformation rates [29].

Our experiments demonstrate that exposure to temperatures ranging from 38 to 40 °C for over 4 h significantly extends the developmental duration of *C. japonicus* larvae and pupae, suggesting that even brief periods of heat stress can hinder development. This outcome mirrors the prolonged developmental period observed in the parasitoid *Psyttalia incisae* Silvestri following heat stress treatment [30]. Moreover, this study reports that the number of mature *C. japonicus* eggs produced decreased by 36.6% and 44.72% following exposure to high-temperature stress during the larval and pupa stages, respectively. This finding highlights potential damage to the insect reproductive system following high-temperature treatment. It is also possible that these treatments accelerate the metabolic rate, causing a more rapid consumption of nutrients stored in the body and less support for reproduction, thereby reducing the number of offspring [31]. In the present study, all high-temperature treatments reduced the longevity of *C. japonicus* females, except for larvae treated at 36 °C for 2–4 h and pupae treated at 36 °C for 2–6 h. The results are consistent with previous studies of other parasitoids, such as *Venturia canescens* Gravenhorst, *Bactrocera cucurbitae* Coquillett, *Callosobruchus chinensis* Linnaeus, *Microplitis manila* Ashmead and *Aphelinus asychis* Walker [32,33,34]. The reduced insect lifespan observed in our study may be caused by high temperatures accelerating the metabolic rate, resulting in damage to intracellular macromolecules by reactive oxygen species. The shortening of lifespan is likely due to an imbalance between insect damage repair and body maintenance [35,36]. The impact of continuous high-temperature stress on insect development and reproduction is highly influenced by factors such as species, developmental stage, and stress duration. In the present study, *C. japonicus* larvae subjected to 3 d of continuous stress at 34 °C experienced reductions in survival rates, and decreased female fecundity. After experiencing stress compared to pupae, the larvae exhibit a relatively minor impact on the survival rate and fecundity. This finding suggests that larvae demonstrate a greater capacity for adaptation to high temperatures compared to pupae. This finding is consistent with a previous report showing reduced adult lifespans and fecundity in *Bradysia odoriphaga* Yang et Zhang following exposure to temperatures above 28 °C. At temperatures exceeding 30 °C, the survival rate of all instars was significantly reduced, and the developmental period was heavily extended [37]. Moreover, the survival rates seemed to consistently exceed 80% across the majority of the treatments in our study (Table 2). This suggests that *C. japonicus* has the potential to live in the areas with even higher temperatures. Therefore, assessing the temperature tolerance of *C. japonicus* in detail, especially in the range above 40 °C, is needed for future studies to evaluate the application of this parasitoid in other tropical regions. A 2004 study found that the average fecundity of *Metopolophium dirhodum* Walker decreased from 51 to 28, and the average female lifespan decreased from 32 to 20 days, under temperatures of 27–33 °C [38]. Our results demonstrate reductions in the lifespan and offspring emergence rates of heat-stressed *C. japonicus*, underscoring the detrimental effects of prolonged exposure to extreme heat conditions (32 °C and 34 °C). Notably, previous works have reported that these adverse impacts are occasionally transgenerational, with parental wasps transmitting unfavorable factors to their offspring, thereby disrupting the trajectories of subsequent generations [39].

The adult body size of insects serves as a crucial indicator of fecundity and fitness [40] and has been extensively studied under various stress conditions. Notably, heat stress has been shown to significantly alter the body size of various insect species, such as *Aphidius colemani* Viereck, *Aphidius gifuensis* Ashmaed, *Encarsia Sophia* Girault and Dodd, and *Harmonia axyridis* Pallas [36,41,42]. This study investigated the effects of short-term heat stress on the larval and pupal stages of *C. japonicus*, revealing that the body length of newly emerged female adults progressively decreased along with increases in temperature and stress duration. For instance, larvae exposed to 40 °C for 4 and 6 h, and pupae exposed to 40 °C for 2, 4, and 6 h, exhibited significantly shorter bodies compared to the CK [43]. However, in most treatments, the variances in body length are relatively minor (Table 2 and Table 4). These findings suggest that heat stress can differentially affect the growth rate and developmental timeline of insects [44,45]. The observed reductions in body size have profound implications for the fitness and reproductive potential of *C. japonicus*, emphasizing the need for further research to elucidate the underlying mechanisms and assess potential consequences for pest control strategies.

The LT50 is an important metric for assessing changes in insect temperature tolerance under different stress conditions [46]. In this study, the LT50 of adult female *C. japonicus* decreased as temperatures increased from 36 °C to 40 °C. This pattern mirrors observations in *Ostrinia furnacalis* Guenée, which exhibited similar responses to heat stress [47]. Further investigation is required to determine whether this decline in heat tolerance is due to the significant evaporation of moisture within parasitoids under high-temperature conditions.

It is vital to assess the functional response of parasitic wasps to evaluate their pest control abilities [48]. In this experiment, the parasitism capabilities of *C. japonicus* at different *P. nigra* densities were calculated after various high-temperature stress treatments, and the results were consistent with the Holling-II model [28]. Compared to the CK, heat treatments resulted in reduced parasitism rates, with the lowest value observed following exposure to 40 °C. This finding supports a previous study that demonstrated reduced parasitism in *Xanthopimpla stemmator* Thunberg under high-temperature conditions [49], suggesting a strong link between the two. This may result from accelerated metabolism influencing mature egg production and motility [50]. Rising temperatures reportedly weaken the parasitism response of *Pimpla turionellae* Linnaeus to its hosts, with reduced oviposition and decreased accuracy in mechanosensory host localization [49]. Our findings show that increases in temperatures ranging from 36 to 40 °C led to a corresponding decrease in the *C. japonicus* instantaneous attack rate (*a****′***), thereby extending total handling time. These findings suggest that high-temperature conditions adversely affect the parasitism behavior of *C. japonicus*, hindering its pest control capabilities.

For the biological control of *P. nigra* using *C. japonicus*, the optimal temperatures of the scale and its parasitoid were similar, as the optimal temperatures of *P. nigra* and *C. japonicus* are 23–27 °C and 24–27 °C, respectively [15,51,52]. Additionally, we found that *C. japonicus* was obviously suppressed when experiencing 34 °C of high-temperature stress over 3 d, whereas the previous study showed that the *P. nigra* egg hatching is inhibited when daily minimum temperatures surpass 29 °C. The result indicates that *C. japonicus* can serve as an effective biological control agent against *P. nigra* in warm regions. Crucially, the timing for the release of *C. japonicus* should be adjusted in accordance with outdoor temperature conditions.

## 5. Conclusions

In conclusion, stress temperature and duration were found to be crucial for the development and reproduction of *C. japonicus*. Exposure to temperatures of 36–40 °C for over 2 h and continuous exposure to 34 °C for more than 3 days adversely affect the insects’ development, survival, and population growth. Similarly, short-term exposures to high temperatures diminished predation on *P. nigra*. Therefore, to optimize the efficacy of *C. japonicus* for the biological control of *P. nigra* in agricultural settings, growers should avoid releasing the parasitoid in temperatures exceeding 34 °C. Applying larval of the parasitoid during cooler temperatures can greatly enhance their field survival, colonization, and pest control effectiveness.

## Figures and Tables

**Table 1:** Two-factor non-parametric ANOVA on the effects of temperature and time of high-temperature stress on larval *Coccophagus japonicus* Compere.

Index	Factor	df	F	*p*
Survival rate (%)	a	3	100.02	<0.001
	b	2	12.13	<0.001
	a × b	6	1.72	0.160
Developmental duration (d)	a	3	30.14	<0.001
	b	2	3.61	0.043
	a × b	6	0.61	0.717
Body length (mm)	a	3	6.76	0.002
	b	2	1.73	0.199
	a × b	6	0.43	0.850
Number of mature eggs (individuals)	a	3	47.01	<0.001
	b	2	2.71	0.087
	a × b	6	0.61	0.719
Lifespan (d)	a	3	30.07	<0.001
	b	2	8.61	0.002
	a × b	6	1.77	0.148 ^1^

^1^ Note: a, b indicate stress temperature and time, respectively.

**Table 2:** Two-factor non-parametric ANOVA on the effects of temperature and time of high-temperature stress on pupal *C. japonicus*.

Index	Factor	df	F	*p*
Survival rate (%)	a	3	28.97	<0.001
	b	2	12.91	<0.001
	a × b	6	1.73	0.156
Developmental duration (d)	a	3	17.43	<0.001
	b	2	7.62	0.003
	a × b	6	1.06	0.412
Body length (mm)	a	3	19.51	<0.001
	b	2	3.42	0.049
	a × b	6	0.95	0.482
Number of mature eggs (individuals)	a	3	58.69	<0.001
	b	2	10.33	<0.001
	a × b	6	1.24	0.320
Lifespan (d)	a	3	36.04	<0.001
	b	2	1.91	0.170
	a × b	6	0.39	0.878 ^1^

^1^ Note: a, b indicate stress temperature and time, respectively.

**Table 3:** Effects of short-term high temperature on larval *C. japonicus*.

Temperature (°C)	Time (d)	Survival Rate (%)	Developmental Duration (d)	Body Length (mm)	Number of Mature Eggs (Individuals)	Lifespan (d)
27	-	98.89 ± 1.11 a	22.39 ± 0.11 d	1.27 ± 0.00 ab	57.17 ± 1.13 a	19.97 ± 0.11 a
36	2	96.39 ± 0.48 a	22.16 ± 0.28 d	1.28 ± 0.01 a	56.03 ± 1.39 a	19.83 ± 0.70 a
	4	91.87 ± 0.87 b	22.56 ± 0.11 cd	1.26 ± 0.01 abc	51.10 ± 2.93 ab	18.88 ± 0.81 ab
	6	92.38 ± 1.19 b	22.49 ± 0.20 cd	1.27 ± 0.01 ab	47.00 ± 2.57 bc	16.47 ± 0.50 cd
38	2	90.07 ± 2.10 bc	22.90 ± 0.15 bcd	1.26 ± 0.01 abc	45.50 ± 1.93 bcd	17.12 ± 0.58 bcd
	4	83.82 ± 1.95 d	23.20 ± 0.27 bcd	1.25 ± 0.01 abc	46.27 ± 2.55 bcd	17.55 ± 0.75 bc
	6	84.57 ± 1.25 d	23.82 ± 0.24 ab	1.24 ± 0.01 bc	41.70 ± 0.99 cde	15.38 ± 0.78 de
40	2	85.55 ± 2.58 cd	23.17 ± 0.24 bcd	1.25 ± 0.02 abc	39.63 ± 1.62 de	16.01 ± 0.65 cd
	4	80.04 ± 1.10 d	23.60 ± 0.36 abc	1.23 ± 0.01 c	38.23 ± 2.91 e	15.10 ± 0.77 de
	6	71.09 ± 3.06 e	24.53 ± 0.82 a	1.23 ± 0.01 c	36.27 ± 2.05 e	13.94 ± 0.14 e
df		9, 20	9, 20	9, 20	9, 20	9, 20
F		22.339	4.68	2.52	11.56	10.51
*p*		<0.001	0.002	0.041	<0.001	<0.001 ^1^

^1^ Note: Data were mean ± SE. Different small letters after data in the same column indicate significant differences (*p* < 0.05), the same as below.

**Table 4:** Effects of short-term high temperature on pupal *C. japonicus*.

Temperature (°C)	Time (d)	Survival Rate (%)	Developmental Duration (d)	Body Length (mm)	Number of Mature Eggs (Individuals)	Lifespan (d)
27	-	98.89 ± 1.11 a	9.06 ± 0.04 cd	1.27 ± 0.00 a	57.17 ± 1.13 a	19.97 ± 0.11 a
36	2	98.89 ± 1.11 a	8.93 ± 0.05 d	1.27 ± 0.01 a	54.63 ± 2.10 a	19.47 ± 0.65 a
	4	94.44 ± 2.94 ab	9.07 ± 0.13 cd	1.26 ± 0.00 a	44.30 ± 1.65 b	19.21 ± 0.55 ab
	6	87.87 ± 3.85 cd	9.30 ± 0.14c	1.25 ± 0.01 ab	43.67 ± 1.64 b	19.03 ± 0.26 ab
38	2	92.22 ± 1.11 bc	9.11 ± 0.09 cd	1.26 ± 0.01 a	44.03 ± 1.45 b	17.60 ± 0.47 bc
	4	88.89 ± 2.94 bcd	9.26 ± 0.04 c	1.24 ± 0.01 ab	40.53 ± 1.84 bc	16.32 ± 0.81 cd
	6	83.33 ± 1.92 de	9.32 ± 0.07 c	1.24 ± 0.01 abc	38.13 ± 0.82 cd	16.69 ± 0.59 cd
40	2	90.00 ±1.92 bcd	9.28 ± 0.04 c	1.22 ± 0.01 bc	40.8 ± 1.81 bc	17.03 ± 0.42 cd
	4	84.44 ± 1.11 de	9.79 ± 0.06 b	1.22 ± 0.01 bc	34.97 ± 1.65 de	15.99 ± 0.69 cd
	6	78.89 ± 2.22 e	10.20 ± 0.08 a	1.21 ± 0.00 c	31.60 ± 2.05 e	15.36 ± 0.48 d
df		9, 20	9, 20	9, 20	9, 20	9, 20
F		22.339	9.203	20.99	5.41	22.96
*p*		<0.001	<0.001	<0.001	<0.001	<0.001 ^1^

^1^ Note: Data were mean ± SE, different small letters after data in the same column indicate significant differences (*p* < 0.05).

**Table 5:** Effects of continuous high-temperature stress on larval *C. japonicus*.

Temperature and Time	Survival Rate (%)	Developmental Duration (d)	Body Length (mm)	Lifespan (d)	Single Female Fecundity (Individuals)
27 °C (CK)	97.97 ± 1.00 a	22.23 ± 0.11 b	1.27 ± 0.00 a	19.97 ± 0.11 a	215.00 ± 4.93 a
Stressed at 32 °C for 3 d	93.55 ± 1.45 a	22.07 ± 0.09 b	1.24 ± 0.01 a	14.46 ± 0.99 b	168.00 ± 13.58 b
Stressed at 34 °C for 3 d	84.67 ± 2.33 b	23.79 ± 0.17 a	1.23 ± 0.01 b	12.14 ± 0.49 c	78.33 ± 3.71 c ^1^

^1^ Note: Data were mean ± SE. Different small letters after data in the same column indicate significant differences (*p* < 0.05).

**Table 6:** Effects of continuous high-temperature stress on pupal *C. japonicus*.

Temperature and Time	Survival Rate (%)	Developmental Duration (d)	Body Length (mm)	Lifespan (d)	Single Female Fecundity (Individuals)
27 °C (CK)	97.97 ± 1.00 a	9.06 ± 0.04 a	1.27 ± 0.00 a	19.97 ± 0.11 a	215.00 ± 4.93 a
Stressed at 32 °C for 3 d	81.11 ± 1.00 b	8.61 ± 0.08 b	1.26 ± 0.01 a	16.51 ± 0.31 b	128.33 ± 16.15 b
Stressed at 34 °C for 3 d	70.00 ± 1.73 c	9.25 ± 0.07 a	1.25 ± 0.01 a	11.37 ± 0.62 c	35.33 ± 2.73 c ^1^

^1^ Note: Data were mean ± SE. Different small letters after data in the same column indicate significant differences (*p* < 0.05).

**Table 7:** Effect of continuous high-temperature stress during the larval stage on the fitness of the offspring of *C. japonicus*.

Temperature and Time	Number of Emerged *C. japonicus* (Individuals)	Developmental Duration (d)	Body Length (mm)	Parasitism Rate (%)
27 °C (CK)	44.33 ± 1.76 a	22.23 ± 0.18 a	1.27 ± 0.01 a	62.22 ± 2.22 a
Stressed at 32 °C for 3 d	42.33 ± 1.86 ab	22.33 ± 0.20 a	1.26 ± 0.01 a	60.00 ± 4.04 a
Stressed at 34 °C for 3 d	37.33 ± 1.45 b	22.74 ± 0.13 a	1.25 ± 0.01 a	54.44 ± 3.93 a ^1^

^1^ Note: Data were mean ± SE. Different small letters after data in the same column indicate significant differences (*p* < 0.05).

**Table 8:** Effect of continuous high-temperature stress during the pupal stage on the fitness of the offspring of *C. japonicus*.

Temperature and Time	Number of Emerged *C. japonicus* (Individuals)	Developmental Duration (d)	Body Length (mm)	Parasitism Rate (%)
27 °C (CK)	44.33 ± 1.76 a	22.23 ± 0.18 a	1.27 ± 0.01 a	62.22 ± 2.22 a
Stressed at 32 °C for 3 d	39.67 ± 0.88 a	22.35 ± 0.24 a	1.26 ± 0.01 a	58.89 ± 2.94 a
Stressed at 34 °C for 3 d	27.67 ± 2.19 b	22.70 ± 0.12 a	1.26 ± 0.01 a	47.78 ± 2.22 b ^1^

^1^ Note: Data were mean ± SE. Different small letters after data in the same column indicate significant differences (*p* < 0.05).

**Table 9:** The female adult LT50 of *C. japonicus* under different high-temperature conditions.

Temperature (°C)	Regression Equation	LT50 (h)	95% Confidence Interval	Standard Error
36	Y = −6.251+ 4.285X	28.78	25.305~33.497	0.741
38	Y = −5.992 + 4.972X	16.04	14.391~17.930	0.792
40	Y = −7.046 + 7.844X	7.91	7.301~8.540	1.083

**Table 10:** Parasitism of *C. japonicus* to different densities of *P. nigra* after adverse temperature stress.

Temperature (°C)	*P. nigra* Density (Individual Per Petri Dish)					
	5	10	15	20	25	30
27	4.67 ± 0.33 aD	8.67 ± 0.67 aC	13.33 ± 0.33 aB	16.00 ± 0.58 aA	17.67 ± 0.67 aA	18.00 ± 1.00 aA
36	3.33 ± 0.33 abD	6.00 ± 0.58 abC	9.00 ± 1.00 bB	10.33 ± 0.88 bAB	11.33 ± 0.88 bAB	12.00 ± 0.58 bA
38	2.67 ± 0.33 bcC	5.33 ± 0.67 bcB	7.33 ± 0.88 bAB	8.67 ± 0.88 bA	9.00 ± 1.00 bcA	8.33 ± 0.33 cA
40	1.33 ± 0.33 cD	2.33 ± 0.33 cCD	2.67 ± 0.67 cCD	3.67 ± 0.67 cBC	4.67 ± 0.67 cAB	5.67 ± 0.67 cA ^1^

^1^ Note: Data are presented as mean ± SE. The different lowercase letters indicate a significant difference at the 0.05 level in the same column; the different uppercase letters showed a significant difference at the 0.05 level in the same line (*p* < 0.05).

**Table 11:** Simulation equations and parameters of Holling-II functional response of *C. japonicus* to *P. nigra* after adverse temperature stresses.

Temperature (°C)	Instant Attack rate (*a′*)	Handing Time (*Th*)	Parasitic Efficiency *a′*/*Th* (Individual·d−1)	Maximum Parasitized Hosts 1/*Th* (Individual·d−1)	Correlation Coefficient (*R*^2^)	Chi-Square (*χ*^2^)
27	1.0163	0.0162	62.74	61.73	0.9973	0.4224
36	0.7518	0.0324	23.20	30.86	0.9977	0.1629
38	0.6198	0.0425	14.58	23.53	0.9885	0.5632
40	0.3027	0.0997	3.04	10.03	0.9894	0.2706

## Data Availability

The data presented in this study are available upon request from the corresponding author. The data are not publicly available due to the uncompleted subject.
